# Adverse childhood experiences are associated with perceived cognitive difficulties among high school students in the United States

**DOI:** 10.3389/fpsyg.2024.1293013

**Published:** 2024-02-05

**Authors:** Ila A. Iverson, Nathan E. Cook, Grant L. Iverson

**Affiliations:** ^1^Department of Global Public Health, Karolinska Institutet, Stockholm, Sweden; ^2^Department of Physical Medicine and Rehabilitation, Harvard Medical School, Boston, MA, United States; ^3^Department of Physical Medicine and Rehabilitation, Spaulding Rehabilitation Hospital, Charlestown, MA, United States; ^4^Mass General for Children Sports Concussion Program, Boston, MA, United States; ^5^Department of Physical Medicine and Rehabilitation, Schoen Adams Research Institute at Spaulding Rehabilitation, Charlestown, MA, United States

**Keywords:** cognitive functioning, adverse child experiences, COVID - 19, adolescents, mental health

## Abstract

**Objective:**

Adverse childhood experiences (ACEs) are associated with mental health and cognitive problems, and mental health problems are associated with perceived cognitive difficulties among adolescents. The unique contribution of ACEs to cognitive difficulties after adjusting for poor mental health is not well understood and represents the purpose of this study.

**Methods:**

The Adolescent Behaviors and Experiences Survey was conducted in 2021 with high school students in the United States. Cognitive difficulty was assessed with: ‘Because of a physical, mental, or emotional problem, do you have serious difficulty concentrating, remembering, or making decisions?’ Four ACEs were examined: sexual violence (lifetime and past 12 months), parental emotional abuse, and parental physical abuse. Students were asked about feeling sad or hopeless (past year), considering suicide (past year), and having poor mental health (past month). Binary logistic regressions examined the association between ACEs and cognitive problems, adjusting for mental health.

**Results:**

Participants were 6,945 students. Students reporting poor mental health were very likely to endorse difficulty concentrating, remembering, or making decisions (girls = 81% and boys = 67%). Cognitive difficulty was uncommon among students who denied poor mental health (girls = 17% and boys = 12%). For boys [*p* < 0.001; *R*^2^ = 0.22] and girls [*p* < 0.001; *R*^2^ = 0.31], after adjusting for mental health problems, independent predictors of cognitive difficulties included parental verbal abuse and physical abuse. For girls, lifetime forced sexual intercourse and sexual violence during the past year were also independently associated.

**Conclusion:**

ACEs are associated with perceived cognitive difficulty in both adolescent girls and boys, even after adjusting for poor mental health.

## 1 Introduction

Adverse childhood experiences (ACEs) are potentially traumatic events that include witnessing violence in the home; having a family member attempt or die by suicide; or personally experiencing maltreatment such as physical abuse, emotional abuse, sexual abuse, or neglect (Gilgoff et al., 2020; Hustedde, 2021; Sherfinski et al., 2021). These early life stressors are associated with poor mental health among youth. For example, ACEs are associated with the development of major depressive disorder in children and adolescents (LeMoult et al., 2020), and **c**hildhood maltreatment, abuse, and neglect are associated with suicidality in children, adolescents, and young adults (Angelakis et al., 2020). In a survey study of high school students, those who reported three or more ACEs (relative to none), had more than eight times greater odds of reporting emotional distress and suicidal ideation (Johnson et al., 2022). ACEs are also associated with cognitive functioning and associated problems experienced by children and adolescents (Johnson et al., 2021). Specifically, a systematic review and meta-analysis concluded that ACEs are associated with lower executive functioning in children and adolescents, as measured with neuropsychological tests (Johnson et al., 2021). Further, in a large survey of high school students, an association between the endorsement of ACEs and poor self-reported academic achievement was found (Duke, 2020).

A substantial percentage of adolescents reported experiencing ACEs during the COVID-19 pandemic (Anderson et al., 2022; Hertz et al., 2023). In one survey of adolescents in the United States, close to one third of youth reported experiencing one or more *additional* adverse experiences during the pandemic (Hertz et al., 2023). There can be a generational pattern of adverse experiences, neglect, and deprivation in that parents with high ACE scores are more likely to engage in child neglect, verbal abuse, and reduced feeding frequency, especially during periods of unpredictability and stress such as the COVID-19 pandemic (Narayan et al., 2021; Rowell and Neal-Barnett, 2022; Arowolo et al., 2023). In an observational study conducted in Germany, increases in paternal stress during the pandemic and parental experience of their own childhood maltreatment were associated with the use of violence against their children (Geprägs et al., 2023). Among high school students in the United States, after adjusting for demographic characteristics, adolescents who reported experiencing four or more ACEs during the pandemic were four times as likely to report poor mental health within the past month and they were 25 times more likely to attempt suicide compared to students who did not report any ACEs (Anderson et al., 2022).

During the COVID-19 pandemic, the United States Centers for Disease Control and Prevention (CDC) conducted a survey of high school students, the Adolescent Behavior and Experiences Survey (ABES) (Rico et al., 2022), to examine disruption and adversity experienced during that unprecedented time. A large percentage of high school students in the United States endorsed having cognitive difficulties (i.e., serious difficulty concentrating, remembering, or making decisions) on that survey (Iverson and Iverson, 2022). That survey also included a question relating to current mental health [i.e., ‘During the past 30 days, how often was your mental health not good? (Poor mental health includes stress, anxiety, and depression)’]. Of those who rated themselves as having poor mental health in the past 30 days, 67% of boys and 81% of girls reported cognitive difficulties (Iverson and Iverson, 2022).

Prior to the COVID-19 pandemic, in 2019, the CDC conducted a survey of high school students called the Youth Risk Behavior Survey (YRBS) (Underwood et al., 2020; Mpofu et al., 2023). Similar to the findings on the ABES, a large percentage of high school students in the United States endorsed having serious difficulty concentrating, remembering, or making decisions because of a physical, mental, or emotional problem on the YRBS in 2019 (Iverson and Iverson, 2022; Onyeaka et al., 2022). Specifically, 45% of girls and 30% of boys reported having cognitive difficulties (Iverson and Iverson, 2022). Of those who reported experiencing depression in the past year, 54% of boys and 69% of girls reported having cognitive difficulties. Of those who reported thoughts of suicide in the past year, 62% of boys and 74% of girls reported having cognitive difficulties. Results from the 2019 YRBS indicated that ACEs were associated with student self-reported cognitive difficulties. Specifically, those with a lifetime history of sexual abuse were likely to endorse having cognitive difficulties (53% of boys and 65% of girls) (Iverson and Iverson, 2022).

Among youth, ACEs have been associated with mental health and cognitive problems and mental health problems are associated with cognitive difficulties. However, the unique contribution of ACEs to problems concentrating, remembering, or making decisions after adjusting for poor mental health is not well understood. The purpose of this study was to examine associations between ACEs and perceived cognitive difficulty in high school students in the United States, during the pandemic, using the 2021 ABES. We hypothesized that ACEs would be independently associated with perceived cognitive difficulty, after adjusting for depression during the past year, suicidality during the past year, and current mental health problems.

## 2 Materials and methods

The YRBS has been conducted by the CDC biennially since 1991, with the intention of assessing risk behavior in high school aged youth in the United States. In 2021, between January and June, the ABES was conducted by the CDC as a one-time, self-administered, anonymous online survey to assess risk behaviors during the COVID-19 pandemic. The ABES employed a similar structure and methodology as the YRBS. There were 23 questions that were additional questions included in the 2021 ABES, including 12 that pertained directly to risk behavior during the pandemic.

The ABES was administered in public and private high schools (schools with grades 9–12) in all 50 states and the District of Columbia in either English or Spanish. The ABES was designed to be completed outside of school on a secure, web-based platform. Students were encouraged to complete the survey in a single sitting, but there was an option available to save their progress and complete the survey later. If students responded to 20 or fewer questions or endorsed the same answer consecutively for more than 15 questions, their data were recorded as “subverted” (i.e., all values were marked as missing except for demographic variables) (Rico et al., 2022). The school response rate was 37.8% (128 of 339 sampled schools participated). The student response rate was 48.0% (7,705 of 16,037 students submitted questionnaires that were eligible after data editing). The overall response rate was 18.1% [i.e., (school response rate) x (student response rate)]. Students enrolled in alternative special education, vocational schools, U.S. Department of Defense, and Bureau of Indian Education were not included. To obtain a nationally representative sample, a three-stage cluster sampling approach was used, and the ABES data was weighted based on sex and grade level of students to account for the oversampling of non-Hispanic Black students and Hispanic students (Rico et al., 2022).

### 2.1 Participants

In the ABES national database, there were data available from 7,705 students. After excluding students with missing data on age, sex, cognitive difficulty, or mental health problems, the final sample included 6,945 students, ages 14–18. Some students in the final sample had missing data on one or more of the ACEs variables. Specifically, 55 students (0.8%) were missing data on sexual assault, 203 students (2.9%) were missing data on sexual violence, 27 students (0.4%) were missing data on parental physical abuse, and 21 students (0.3%) were missing data on parental emotional abuse. The final sample included 3,678 girls (53%) and 3,267 boys (47%). The age composition of the sample was as follows: 14 years old, *n* = 816 (11.7%); 15 years old, *n* = 1,746 (25.1%); 16 years old, *n* = 1,784 (25.7%); 17 years old, *n* = 1,633 (23.5%); and 18 years old, *n* = 966 (13.9%). Students’ self-reported racial/ethnic identities were as follows: 3,186 students identified as White (45.9%), 1,018 students identified as Black (14.7%), 320 students identified as Asian (4.6%), 1,836 students identified as Hispanic or multiple Hispanic ethnicities (26.4%), and 585 students reported they were not represented by these racial/ethnic categories (8.4%).

### 2.2 Survey questions and combined variables

Broadly, there were six categories of behavior addressed in the ABES: (a) behaviors that contribute to unintentional injury and violence, (b) tobacco product use, (c) alcohol and other drug use, (d) sexual behaviors, (e) dietary behaviors, and (f) physical inactivity. The 12 questions relating to behavior and experiences during the pandemic encompass the following experiences and behaviors: (a) increased use of alcohol and drugs, (b) economic and food insecurity, (c) abuse by a parent, (d) poor mental health, (e) school and social engagement, and (f) use of telemedicine. The ABES questionnaire, data, and documentation are publicly accessible and available on the CDC website (https://www.cdc.gov/healthyyouth/data/abes.htm).

The purpose of this study was to examine the association between ACEs and self-reported cognitive problems among youth during the pandemic. We examined the cognition question of interest (i.e., ‘Because of a physical, mental, or emotional problem, do you have serious difficulty concentrating, remembering, or making decisions?’ Response options: ‘Yes’ or ‘No’) in relation to four ACE variables. The wording of the question of interest positions cognitive problems as being secondary to a physical, mental, or emotional problem. Some students may attribute their cognitive problems with concentration, memory, and decision making to mental health problems (e.g., depression, suicidality), psychiatric conditions, neurodevelopmental conditions, emotional dysregulation, or medical problems (e.g., traumatic brain injury, chronic illness).

The four questions relating to ACEs used in this study are listed in Table 1. Two of these questions relate to forced sexual intercourse or sexual violence. These variables have been included verbatim in previous versions of the YRBS. Two of the questions are about parental physical or emotional abuse during the pandemic and were added for the first time in the 2021 ABES. In Table 1, all questions are denoted by QN#. For binary variables QN refers to coding the response options as ‘1’ and ‘2’ for ‘Yes’ and ‘No’ respectively. An ACEs index was created by summing responses to the four questions involving ACEs.

**Table 1:** Adolescent behavior and experiences survey (ABES) questions.

Forced sexual intercourse. QN19. Have you ever been physically forced to have sexual intercourse when you did not want to? Response options: (a) Yes, (b) No. Sexual violence. QN20. During the past 12 months, how many times did **anyone** force you to do sexual things that you did not want to do? (Count such things as kissing, touching, or being physically forced to have sexual intercourse). Response options: (a) 0 times, (b) 1 time, (c) 2 or 3 times, (d) 4 or 5 times, (e) 6 or more times. Students who endorsed response options b-e were coded as experiencing sexual violence. Parental emotional abuse during pandemic. QN105. During the COVID-19 pandemic, how often did a parent or other adult in your home swear at you, insult you, or put you down? Response options: (a) Never, (b) Rarely, (c) Sometimes, (d) Most of the Time, (e) Always. Students who endorsed response options b-e were coded as experiencing parental emotional abuse. Parental physical abuse during pandemic. QN106. During the COVID-19 pandemic, how often did a parent or other adult in your home hit, beat, kick, or physically hurt you in any way? Response options: (a) Never, (b) Rarely, (c) Sometimes, (d) Most of the Time, (e) Always. Students who endorsed response options b-e were coded as experiencing parental physical abuse. Sad or hopeless. QN25. During the past 12 months, did you ever feel so sad or hopeless**almost every day for 2 weeks or more in a row that you stopped doing some usual** activities? Response options: (a) Yes, (b) No. Considered suicide. QN26. During the past 12 months, did you ever **seriously** consider attempting suicide? Response options: (a) Yes, (b) No. Current Mental Health: QN85. During the past 30 days, how often was your mental health not good? (Poor mental health includes stress, anxiety, and depression.) Response options: (a) Never, (b) Rarely, (c) Sometimes, (d) Most of the time, and (e) Always. Endorsing (d) or (e) was coded as ‘poor mental health.’ Response options: (a) Never, (b) Rarely, (c) Sometimes, (d) Most of the Time, (e) Always. Students who endorsed response options (d) or € were coded as poor current mental health. Cognitive Difficulty. QN115. Because of a physical, mental, or emotional problem, do you have serious difficulty concentrating, remembering, or making decisions? Response options: (a) Yes, (b) No.

These questions were derived from the 2021 ABES data user’s guide, which can be found here: https://www.cdc.gov/healthyyouth/data/abes.htm. Most binary options were coded for analyses as 0 = ‘No’ and 1 = ‘Yes.’

### 2.3 Statistical analyses

Unadjusted and adjusted associations were examined using binary logistic regressions with self-reported cognitive difficulty as the dichotomous dependent variable. The analyses were stratified by sex. Four variables relating to ACEs and three variables relating to mental health status were the predictor variables (see in Table 1). Odds ratios (OR) above 1.0 with a 95% confidence interval (CI) not including 1.0 reveal predictors that are associated with greater odds of endorsing cognitive difficulty.

A multivariable logistic regression, stratified by gender, was conducted including all ACEs in the same model to examine the magnitude of their associations after adjusting for depression within the past year, suicidality within the past year, and current mental health [i.e., ‘During the past 30 days, how often was your mental health not good? (Poor mental health includes stress, anxiety, and depression)’]. The ORs are interpreted in the same manner as described above, although they reflect the increase in odds of endorsing cognitive difficulty after adjusting for all other variables in the model.

## 3 Results

**Figure 1:**
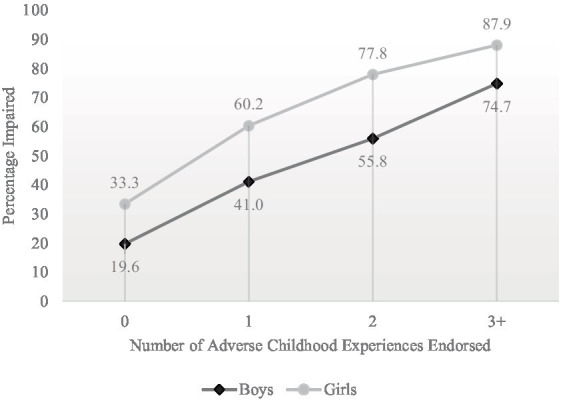
Percentages of students endorsing perceived cognitive difficulty stratified by the number of adverse childhood experiences endorsed. Sample sizes for boys were as follows: 0 = 1,654, 1 = 1,169, 2 = 369, 3 + =75. Sample sizes for girls were as follows: 0 = 1,251, 1 = 1,504, 2 = 608, 3 + =315.

The results of subgroups of youth who reported ACEs variables and cognitive problems with concentration, memory, and decision making are presented in Table 2. These results are stratified by sex due to the difference in reporting of cognitive difficulty between boys and girls. Girls (56.3%) were significantly more likely to endorse cognitive difficulty compared to boys [32.6%; *χ*^2^ (1) = 392.71, *p* < 0.001]. A high percentage of boys and girls who reported experiencing parental emotional abuse during the pandemic also reported having cognitive difficulties (i.e., 46.3% of boys and 68.9% of girls). In addition, a high percentage of boys and girls who reported a lifetime history of sexual abuse also reported having cognitive difficulties (i.e., 64.9% of boys and 82.8% of girls). There was a strong association between the number of ACEs endorsed and perceived cognitive difficulties, as illustrated in Figure 1, such that youth reporting increasing numbers of ACEs were considerably more likely to endorse cognitive problems for both girls (*χ*^2^ (3) = 521.60, *p* < 0.001) and boys (*χ*^2^ (3) = 315.76, *p* < 0.001). For example, rates of cognitive difficulty among youth reporting no ACEs (33.3% for girls; 19.6% for boys) were considerably lower compared to youth reporting 3 or all 4 ACEs (87.9% for girls; 74.7% for boys).

**Table 2:** Percentages of high school students endorsing cognitive difficulty stratified by mental health status and adverse childhood experiences.

	Percentages of the subgroups endorsing Perceived cognitive difficulty					
	Total sample		Boys		Girls	
**Group/subgroup**	*n*	%	*n*	%	*n*	%
Total sample	6,945	45.2	3,267	32.6	3,678	56.3
Poor mental health (past 30 days)	2,130	77.2	622	67.2	1,508	81.3
Sad or hopeless	3,062	71.3	1,027	61.7	2,035	76.1
Considered suicide	1,400	81.1	450	71.8	950	85.5
No poor mental health (past 30 days)	1,514	11.8	1,090	11.0	424	13.9
Not sad or hopeless	3,864	24.4	2,233	19.2	1,631	31.5
Did not consider suicide	5,506	35.8	2,806	26.3	2,700	45.7
Never or rarely mental health variables	2,403	13.6	1,607	12.0	796	16.7
None of three mental health variables	1,353	9.5	992	9.1	361	10.8
**Adverse childhood experiences**						
Forced sexual intercourse (lifetime)	502	79.5	94	64.9	408	82.8
Sexual violence (past year)	689	76.9	131	62.6	558	80.3
Parental emotional abuse during pandemic	3,851	59.8	1,551	46.3	2,300	68.9
Parental physical abuse during pandemic	850	70.2	379	57.5	471	80.5

These are the percentages of youth who said “yes” to the following question (Q115): “Because of a physical, mental, or emotional problem, do you have serious difficulty concentrating, remembering, or making decisions?” Poor mental health is the percentage of students who reported that their mental health was most of the time or always not good (including stress, anxiety, and depression, during the 30 days before the survey). ‘No poor mental health’ refers to youth who said ‘never’ to “during the past 30 days, how often was your mental health not good?” (Q85). ‘Never or rarely mental health variables’ are youth who said ‘never’ or ‘rarely’ to Q85 and said ‘No’ to depression (Q25) and suicidality (Q26).

Binary logistic regressions were used to examine the associations between cognitive difficulty, mental health variables, and ACEs. Both the unadjusted and adjusted results are presented in Table 3, stratified by sex. In the unadjusted analyses, all four ACEs variables were significantly associated with cognitive difficulties for both boys and girls (ORs ranging from 3.2–4.3; Table 3). In the multivariable model, after adjusting for current mental health, depression, and suicidality, parental emotional and physical abuse were independently associated with cognitive problems for both boys and girls (ORs ranging from 1.4–1.9). For girls, sexual assault and sexual violence were also independently associated with cognitive problems (ORs ranging from 1.4–1.5).

**Table 3:** Logistic regressions predicting perceived cognitive difficulty by gender.

	Multivariable model				Adjusted			Unadjusted		
						95% CI			95% CI	
Girls	*B*	*SE*	Wald	*p*	OR	Lower	Upper	OR	Lower	Upper
Poor mental health	1.161	0.092	159.888	<0.001	3.192	2.666	3.821	6.817	5.835	7.965
Sad or hopeless	1.006	0.089	127.373	<0.001	2.734	2.296	3.256	6.926	5.986	8.015
Considered suicide	0.752	0.122	38.248	<0.001	2.121	1.671	2.692	6.990	5.748	8.501
Forced sexual intercourse (lifetime)	0.418	0.175	5.728	0.017	1.520	1.079	2.141	4.333	3.319	5.656
Sexual violence (past year)	0.325	0.140	5.420	0.020	1.384	1.053	1.820	3.954	3.171	4.930
Parental emotional abuse during pandemic	0.648	0.087	55.224	<0.001	1.911	1.611	2.267	4.095	3.553	4.721
Parental physical abuse during pandemic	0.410	0.148	7.717	0.005	1.507	1.128	2.012	3.692	2.909	4.684

					Adjusted			Unadjusted		
						95% CI			95% CI	
Boys	*B*	*SE*	Wald	*p*	OR	Lower	Upper	OR	Lower	Upper
Poor mental health	1.015	0.113	80.364	<0.001	2.758	2.210	3.443	6.328	5.236	7.647
Sad or hopeless	1.188	0.100	141.519	<0.001	3.279	2.696	3.987	6.803	5.774	8.017
Considered suicide	0.750	0.139	29.302	<0.001	2.116	1.613	2.777	7.140	5.719	8.913
Forced sexual intercourse (lifetime)	0.144	0.285	0.255	0.614	1.155	0.660	2.202	3.983	2.591	6.124
Sexual violence (past year)	0.262	0.237	1.224	0.268	1.300	0.817	2.067	3.738	2.602	5.368
Parental emotional abuse during pandemic	0.636	0.094	45.687	<0.001	1.889	1.571	2.272	3.403	2.915	3.972
Parental physical abuse during pandemic	0.364	0.141	6.703	0.010	1.439	1.093	1.896	3.248	2.610	4.043

See Table 1 for definitions of these variables. CI = confidence interval and OR = odds ratio. Multivariable model results for girls (*n* = 3,495): *χ*^2^ (7) = 1,134.937, *p* < 0.001, Cox and Snell *R*^2^ = 0.279, Nagelkerke *R*^2^ = 0.374. Multivariable model results for boys (*n* = 3,179): *χ*^2^ (7) = 790.634, *p* < 0.001, Cox & Snell *R*^2^ = 0.221, Nagelkerke *R*^2^ = 0.309. Adjusted ORs are from the multivariable model and unadjusted ORs are from separate regression models for each individual predictor.

## 4 Discussion

A remarkably high proportion of high school students in the United States reported serious difficulty concentrating, remembering, or making decisions due to a physical, mental, or emotional problem (i.e., 56% of girls and 36% of boys). After statistically adjusting for mental health problems within the past month, depression in the past year, and suicidality in the past year, several ACEs were independently associated with cognitive problems with concentration, memory, and decision making among adolescents. For both girls and boys, parental physical and emotional abuse were independently associated with cognitive problems. For girls, forced sexual intercourse and sexual violence were also independently associated.

Self-reported cognitive difficulties are commonly experienced by people with neurodevelopmental, psychiatric, and neurological conditions, and subjectively-experienced cognitive difficulty is one of the diagnostic criteria for attention-deficit/hyperactivity disorder (ADHD), major depressive disorder, and generalized anxiety disorder (American Psychiatric Association, 2013). Therefore, it is not surprising, in the present study, that there was a strong association between the experience of poor mental health, depression, and/or suicidality and the endorsement of cognitive problems. As seen in Table 2, over three-fourths of high school students who endorsed poor current mental health, depression, or suicidality reported serious cognitive problems. In contrast, only 10% of students who never experienced mental health problems within the past 30 days and denied depression or suicidality in the past year endorsed cognitive difficulty (see Table 2).

It is well established in the literature that exposure to ACEs confers an elevated risk for psychiatric and mental health diagnoses (e.g., depression, PTSD, suicidality) (Bomysoad and Francis, 2020; Nelson et al., 2020; Lowthian et al., 2021), and prior studies reported that youth and young adults exposed to ACEs had greater COVID-19 related stress and worse mental health during the pandemic (Stinson et al., 2021; Alradhi et al., 2022). ACEs were a significant predictor for an increase in depressive symptoms during the pandemic (Clemens et al., 2022). Further, childhood adversity is associated with negative effects on brain development, such as reduced prefrontal cortex, hippocampal, and amygdala volumes (McLaughlin et al., 2019), suggesting potential neurobiological contributions to perceived and self-reported cognitive difficulties. Therefore, it is reasonable to assume that ACEs, mental health problems, and perceived cognitive difficulties are likely inter-related—as illustrated in this study.

For both boys and girls, even after adjusting for mental health, there was a significant association between physical and emotional abuse and cognitive difficulty. There is evidence that there was an increase in physical abuse and neglect against children during the COVID-19 pandemic (Ezeokoli et al., 2023; Fredin et al., 2023; Thierry et al., 2023). Physical and emotional abuse, and other early life stressors in childhood, can confer an elevated risk for negative mental health outcomes such as the development of major depressive disorder (LeMoult et al., 2020), marijuana and cocaine use during adolescence and early adulthood (Scheidell et al., 2018), and worse academic performance and outcomes (Romano et al., 2015; Tanaka et al., 2015). In the present study, for girls, after adjusting for mental health, there was a significant association between sexual abuse and cognitive difficulty. Youth who experience sexual assault have been found to receive lower grades in school, drop out of school at a higher rate, and have lower levels of education than those who did not experience childhood sexual assault (Ochoa and Constantin, 2023). Several possible factors that might influence or mediate the association between academic achievement and sexual, physical, and emotional abuse have been identified, such as negative future orientation (Ochoa and Constantin, 2023), perceived stress (Bhattarai et al., 2023), and self-esteem (Bhattarai et al., 2023).

### 4.1 Limitations

There are several limitations in this study. First, causal inferences cannot be drawn about the association between ACEs and cognitive difficulty because a cross-sectional survey design was used. Second, only a single question in the ABES assessed cognitive problems with concentration, memory, and decision making, and rating scales composed of multiple items might better assess the construct of perceived cognitive difficulty. Third, the ABES was administered online in students’ homes during the COVID-19 pandemic, and it had a low overall response rate (i.e., 18%)—which increases the likelihood of sampling bias. Fourth, we cannot draw inferences regarding how these high school students would perform on standardized neuropsychological tests. That said, a systematic review and meta-analysis concluded that ACEs are associated with lower scores on neuropsychological tests of executive functioning in children and adolescents (Johnson et al., 2021). Fifth, we cannot draw inferences from this survey study relating to the students’ brain development. The neuroimaging literature, however, suggests that childhood adversity is associated with negative effects on brain development, such as reduced prefrontal cortex, hippocampal, and amygdala volumes (McLaughlin et al., 2019).

Finally, and importantly, given the structure and wording of the survey question, it is not possible to dissociate mental health problems from perceived cognitive difficulty. We assume that a large percentage of students attributed their cognitive difficulties to, or experienced them in clear association with, emotional health problems. Some students, however, might have a neurodevelopmental condition (e.g., ADHD), a traumatic brain injury, neurological disorder, or general medical problem that they considered to be associated with their cognitive difficulties. In the multivariable model we statistically adjusted for sadness and hopelessness in the past year, suicide ideation in the past year, and poor mental health in the past 30 days—and these adjustments substantially reduced the magnitude of the association between perceived cognitive difficulty and the ACEs (see Table 3). However, the associations remained practically and clinically meaningful.

### 4.2 Conclusions and directions for future research

It is well established in the literature that ACEs are associated with a broad range of physical and mental health conditions and problems in adolescents and in adults. In the present study, there was an association between ACEs and perceived cognitive problems with concentration, memory, and decision making among high school students in the United States during the COVID-19 pandemic, even after statistically adjusting for poor mental health in the past month, depression in the past year, and suicidality in the past year. The mechanisms underlying the association between ACEs and perceived cognitive difficulty are unknown, likely multifactorial, and possibly neurodevelopmental, neurobiological, and psychological (Oh et al., 2018; McLaughlin et al., 2019; Petruccelli et al., 2019). Greater and more effective so-called “upstream” interventions at the level of the home, school, and community are needed to reduce the potential negative “downstream” cognitive, academic, and emotional health problems associated with ACEs.

It is possible that some mechanisms underlying the association between perceived cognitive difficulty and ACEs are psychological and modifiable. Assuming so, some of those beliefs might be amenable to belief-change interventions (Schleider and Weisz, 2018). Innovative single-session interventions have been used in several ways to promote better emotional health among adolescents (Schleider and Weisz, 2018; Ching et al., 2023; Loades and Schleider, 2023). It is possible that single-session interventions could be developed to promote cognitive self-efficacy in children and adolescents, particularly those who have experienced ACEs.

## Data availability statement

Publicly available datasets were analyzed in this study. This data can be found at: https://www.cdc.gov/healthyyouth/data/abes.htm.

## Ethics statement

The present study involves secondary analyses of publicly available fully-deidentified data, and thus is considered not human subjects research. The original studies involving humans were approved by the United States Center for Disease Control and Prevention. The Institutional Review Board of the United States Center for Disease Control and Prevention approved the protocol for the Adolescent Behavior and Experiences Survey (ABES). The studies were conducted in accordance with the local legislation and institutional requirements. Parental permission was granted, and students agreed to participate. See Rico et al. (2022).

## Author contributions

II: Conceptualization, Formal analysis, Writing – original draft, Writing – review & editing. NC: Methodology, Supervision, Validation, Writing – review & editing. GI: Conceptualization, Data curation, Formal analysis, Funding acquisition, Investigation, Project administration, Resources, Supervision, Validation, Writing – original draft, Writing – review & editing.
